# Trends in Pharmacy Practice Communication Research

**DOI:** 10.3390/pharmacy6040127

**Published:** 2018-12-05

**Authors:** Sofia Kälvemark Sporrong, Susanne Kaae

**Affiliations:** Department of Pharmacy, Faculty of Health and Medical Sciences, University of Copenhagen, 2100 Copenhagen, Denmark; sofia.sporrong@sund.ku.dk

Communication is a crucial aspect of pharmacy practice—in community pharmacies and in other health care settings. The communicative role of pharmacists and other pharmacy staff is an important part of, e.g., dispensing, pharmaceutical care, and other counselling services provided to patients. It is through appropriate communication that patients’ needs can be assessed and information, education, and advice can be given, in this way working towards a rational use of medicine. In addition, effective inter-professional communication with other health care professionals is central to health outcomes of patient treatments.

Communication is a highly complex area, dealing with not only the transmission of content but also interpersonal relationships, social processes, etc. Barriers and facilitators to communication are numerous, including psychological, socio-economic, cognitive, and environmental factors. Research in pharmacy practice communication can enable the development of the skills, tools, and processes to make patient encounters and other interactions as optimal as possible.

The Special Issue ‘Communication in Pharmacy Practice’ was thus launched to help improve communication practices by increasing our knowledge on different aspects of communication in pharmacy practice. From the articles we have received for this Special Issue, it is clear that pharmacy communication is a subject that is investigated globally and from many perspectives. The overall pharmacy communicational themes investigated and discussed include the following: Communication between health care professionals and elements of communication between pharmacists and patients, in the context of both prescription and OTC medicines; and factors impacting these types of communication. A central focus in this Special Issue is the specific need to further develop direct, face-to-face communication between pharmacy staff and patients/consumers.

With regard to communication between pharmacists and patients, the need to focus counselling on the patient’s perspective is emphasized by several authors. When patients are involved in their own care and understand their plan of care, they are better able to manage their conditions. Naughton writes [1]:

Only when pharmacists have a holistic understanding of an individual patient, including their experience of illness and medication, can they effectively assess appropriateness, safety, efficacy, and adherence to medications and develop realistic treatment plans.

This is supported by Hawes, who discusses how pharmacists’ exploration of cultural aspects, including health and illness beliefs of the patient along with the patient’s attitudes and practices, should be the basis for counselling. In her article about patient education on oral anticoagulation, she concludes that *“the teaching should be tailored to each patient”* [2]. 

As part of this patient-centred approach, Olufemi-Yusuf et al. explored patients’ perceptions of asthma, asthma treatment, and pharmacist roles in order to optimize the design of patient-centred interventions in pharmacy care and improve care for asthma patients [3].

Other authors also investigated aspects influencing pharmacy staff-patient communication such as perception of roles, organizational aspects including the need for sufficient time, privacy, and use of adequate registration systems, and the importance of education for pharmacy staff [4,5,6].

Some specific technologies to develop patient–pharmacist communication are presented in this issue, e.g., pictograms, automated phone calls, and the use of videos to communicate information on inhaler techniques [7,8,9]. For example, Kanji et al. studied pictograms as a technique for pharmacy communication in the presence of language barriers and identified several challenges in patients’ understanding of the pictograms [7].

These signs [pictograms] on their own might not be enough to guarantee appropriate patient information and the expected medication usage.

A study on inhaler instruction videos found that, in addition to watching videos, participants asked for feedback from health care professionals to check their inhalation technique. Hence, it seems that technologies used on their own appear often to be of limited support for patients [9].

One basis for improving pharmacist–patient communication is to determine how the communication between the pharmacists and other health care professionals is conducted. The study by De Bock et al. investigating the implementation of medication reviews across hospital and community pharmacy sections found that discharge notes from hospital to community pharmacists facilitated pharmaceutical care counselling in the community pharmacy [10]. Noss et al. also highlighted the importance of adequate inter-professional communication for the monitoring or use of alternative therapy agents to avoid drug–drug interactions [11].

There are many challenges to communication in pharmacy practice, and many are highlighted in this Special Issue on pharmacy communication. Some appropriate and sustainable solutions are described, but in many cases, these seem to remain on a theoretical level. Seubert et al. tried to overcome this gap by developing specific tools for community pharmacies to overcome identified challenges at the pharmacy counter [12]. The tools were based on the existing literature and other types of empirical material; however, they still need to be implemented in practice. 

Many relevant pharmacy practice communicational aspects have now been added to our knowledge, but even more research is needed. This is especially true for research dealing with how to overcome identified communication challenges in practice to ultimately help patients achieve better treatment outcomes.
